# Delivery cost of the first public sector introduction of typhoid conjugate vaccine in Navi Mumbai, India

**DOI:** 10.1371/journal.pgph.0001396

**Published:** 2023-01-04

**Authors:** Dayoung Song, Sarah W. Pallas, Rahul Shimpi, N. Ramaswamy, Pradeep Haldar, Pauline Harvey, Pankaj Bhatnagar, Arun Katkar, Niniya Jayaprasad, Abhishek Kunwar, Sunil Bahl, Win Morgan, Raymond Hutubessy, Kashmira Date, Vittal Mogasale

**Affiliations:** 1 Policy and Economic Research Department, International Vaccine Institute, Seoul, Republic of Korea; 2 Global Immunization Division, Centers for Disease Control and Prevention, Atlanta, Georgia, United States of America; 3 World Health Organization, India Country Office, New Delhi, India; 4 Navi Mumbai Municipal Corporation, Navi Mumbai, India; 5 Ministry of Family Health and Welfare, Government of India, New Delhi, India; 6 World Health Organization, Regional Office for South-East Asia, New Delhi, India; 7 Levin and Morgan LLC, Bethesda, MD, United States of America; 8 Department of Immunization, Vaccines and Biologicals, World Health Organization, Geneva, Switzerland; 9 Policy and Economic Research Department, International Vaccine Institute, Republic of Korea; Heidelberg University, GERMANY

## Abstract

Navi Mumbai Municipal Corporation (NMMC), a local government in Mumbai, India, implemented the first public sector TCV campaign in 2018. This study estimated the delivery costs of this TCV campaign using a Microsoft Excel-based tool based on a micro-costing approach from the government (NMMC) perspective. The campaign’s financial (direct expenditures) and economic costs (financial costs plus the monetized value of additional donated or existing items) incremental to the existing immunization program were collected. The data collection methods involved consultations with NMMC staff, reviews of financial and programmatic records of NMMC and the World Health Organization (WHO), and interviews with the health staff of sampled urban health posts (UHPs). Three UHPs were purposively sampled, representing the three dominant residence types in the catchment area: high-rise, slum, and mixed (high-rise and slum) areas. The high-rise area UHP had lower vaccination coverage (47%) compared with the mixed area (71%) and slum area UHPs (76%). The financial cost of vaccine and vaccination supplies (syringes, safety boxes) was $1.87 per dose, and the economic cost was $2.96 per dose in 2018 US dollars. Excluding the vaccine and vaccination supplies cost, the financial delivery cost across the 3 UHPs ranged from $0.37 to $0.53 per dose, and the economic delivery cost ranged from $1.37 to $3.98 per dose, with the highest delivery costs per dose in the high-rise areas. Across all 11 UHPs included in the campaign, the weighted average financial delivery cost was $0.38 per dose, and the economic delivery cost was $1.49 per dose. WHO has recommended the programmatic use of TCV in typhoid-endemic countries, and Gavi has included TCV in its vaccine portfolio. This first costing study of large-scale TCV introduction within a public sector immunization program provides empirical evidence for policymakers, stakeholders, and future vaccine campaign planning.

## Background

Typhoid fever is a major health problem in many low- and middle-income countries (LMICs) with global burden estimated between 11 and 21 million cases annually [1]. It is a significant problem in India, particularly affecting young children [2]. Though most typhoid fever cases can be treated with antibiotics, there is an increasing problem of antimicrobial resistance and the spreading of multidrug-resistant strains [3, 4]. Especially in LMICs, water and sanitation measures have paramount importance in typhoid fever control in the long term; however, vaccination is a complementary approach that can help in the short- and medium-term [5]. Typhoid conjugate vaccines (TCV) are an important recent innovation in such control measures.

After the first TCV was licensed in India in 2013 (Typbar-TCV manufactured by Bharat Biotech, India), multiple initiatives have sought to build the evidence base to support policy and financing for TCV introduction [1]. Modeling studies have shown that TCVs have the potential to substantially reduce typhoid disease burden and are likely to be cost-effective in countries with high disease burden [6, 7]. The World Health Organization’s Strategic Advisory Group of Experts (SAGE) on Immunization, an independent advisory committee that advises WHO on immunization policy, recommended TCV use in October 2017 after reviewing all evidence, thereby initiating a change in global typhoid vaccination policy [8]. This was followed by WHO prequalification of the first TCV (Typbar-TCV, Bharat Biotech, India) in December 2017 and the release of an updated WHO typhoid vaccine position paper recommending the programmatic use of TCV in the immunization program for controlling endemic disease [9]. In parallel, Gavi, the Vaccine Alliance, committed financing of $85 million for TCV introduction in eligible countries [10], which has resulted in the approval of Typbar-TCV use in several countries to date [11]. In December 2020, a second TCV (TYPHIBEV, manufactured by Biological E, India) received WHO prequalification, potentially increasing the vaccine supply available for LMICs [12]. Thus, TCVs are expected to play an increasing role in typhoid fever prevention and control in the near future.

Although TCVs hold much promise based on modeling studies, it remains important to evaluate their effectiveness and delivery costs empirically in real-world settings, as has begun to emerge with TCV use in some settings [13]. India is known to be highly endemic for typhoid, thus policymakers there need robust evidence on vaccination feasibility, effectiveness, cost of vaccine delivery, and cost-effectiveness that demonstrates the value of TCV introduction in India. In 2018, the first public sector campaign using a TCV (Typbar-TCV, Bharat Biotech, India) was conducted by the Navi Mumbai Municipal Corporation (NMMC), the local governing body, in Navi Mumbai, Maharashtra, India. The details, decision making, and implementation of the TCV introduction are described in a previous publication [14]. Along with TCV introduction, an evaluation program consisting of enteric fever surveillance, a vaccine effectiveness study, delivery cost estimation, typhoid fever cost of illness study, and cost-effectiveness analysis was implemented by international and national organizations in partnership with NMMC.

This manuscript presents the program cost analysis of this campaign, offering the first empirical evidence on the detailed delivery costs of a TCV in an applied setting. These results can be of use for other settings in India and potentially in similar settings elsewhere to consider the resource requirements for TCV campaigns. In combination with forthcoming vaccine effectiveness and enteric fever cost of illness results, these TCV delivery costs can be used to estimate the cost-effectiveness of TCV introduction in Navi Mumbai.

### Study setting and vaccination campaign program activities

Navi Mumbai is the extension of the suburbs of Mumbai within Maharashtra State in India and is divided into 14 administrative areas with a total population of 1.12 million [14]. NMMC is responsible for 8 of 14 administrative areas in Navi Mumbai; these 8 areas are subdivided into 22 urban health posts (UHP). The TCV campaign in Navi Mumbai took place from July to August 2018, covering the catchment areas of 11 of the 22 UHPs and targeted children aged 9 months up to 15 years who resided in these 11 UHPs. The total target population was estimated at 159,831 as of 2018 based on recent polio vaccination campaign microplans and coverage results (WHO/India, personal communication). The campaign was implemented primarily through outreach sessions at fixed post community locations during weekends and public holidays over a 6-week period, with vaccination at health facilities during the weekdays for children who missed the weekend/holiday campaign days and three days of mop-up outreach vaccination sessions at the end of the campaign; details of campaign implementation have been reported previously [14].

### Cost analysis methods

#### Ethics statement

The delivery cost estimation of TCV in Navi Mumbai presented here is part of a research protocol named “Economic analysis of typhoid conjugate vaccine use in Navi Mumbai, India” which obtained approval from relevant ethics committees. This included the Institutional Review Board of the International Vaccine Institute (IVI), Seoul, Korea (Ref. No: IVI IRB# 2018–003) and the Human Ethical Committee of the Regional Medical Research Center. The delivery cost estimation component of research presented in this manuscript did not involve collection of data on human subjects and therefore consent process was not required.

#### Sampling for data collection

The 11 UHPs included in the campaign covered a mix of residential, commercial, and industrial areas, including “high-rise” apartment building complexes occupied by higher-income residents, slum areas, slum resettlement areas (low-rise apartment buildings), and a quarry with migrant workers [14]. Three UHPs out of 11 UHPs were purposively selected to represent each of three types of UHPs involved in the campaign based on dominant residence type in the catchment area: high-rise (i.e., high income) (1 UHP total), slum (3 UHPs total), and mixed (mix of high-rise and slum areas, 7 UHPs total).

#### Data collection

The incremental cost analysis was conducted from the local government payers’ perspective. The time frame for the analysis was from the start of planning activities for the campaign through the conclusion of the campaign implementation including mop-up activities (December 2016-August 2018). Costs were collected in January 2019 in 2018 Indian rupees (INR). The data collected included both financial costs (i.e., monetary outlays by NMMC) and economic costs (i.e., financial costs plus the value of existing, in-kind, and donated resources, including any expenses covered by external partners) that were incremental (i.e., additional) to the existing immunization and public health program. A Microsoft Excel-based tool was developed to estimate the vaccination campaign costs using a micro-costing approach (S1 Tool). The activities involved in the vaccination campaign were categorized into eight main program activity groups: planning and preparation, microplanning, training, sensitization (i.e., providing information to officials and community leaders), social mobilization, service delivery, supervision and monitoring, and AEFI management. Each group had sub-group activity categories (S1 Table). Information on quantities and prices of resources used for campaign-related activities were collected by evaluation co-investigators from IVI (DS, VM), with technical assistance from CDC (SP), from NMMC and WHO financial and programmatic records, consultation with NMMC and WHO-India personnel involved in the activities and purchase decisions, and through semi-structured interviews with the Medical Officer (MO) and other staff members involved in campaign implementation at each of the three sampled UHPs that asked about time and other resources used by program activity following the structure of the Excel tool (S1 Tool). The costs of buildings and overhead costs of NMMC and UHPs were excluded from the analysis. The cost to the WHO India Country Office of hiring a private public relations firm for social mobilization materials development and media monitoring and engagement was also excluded from the main analysis as this cost was not reported during data collection and analysis, and was only identified during post-hoc results review. Therefore, a post-hoc sensitivity analysis including the cost of this outsourced activity was presented separately. The costs borne by WHO-India were included as economic costs since the local WHO-India staff were part of the planning and implementation of the TCV campaign in Navi Mumbai.

For each campaign activity, personnel costs, allowance costs, supply and material costs, and other direct costs were collected (Table 1). For personnel costs, NMMC and UHP staff salaries with total benefits package were collected by cadre based on NMMC official salary scales, which was validated with NMMC and UHP staff; external partner (e.g., WHO) staff salaries and benefits were collected from the respective partner organizations. Time spent on each activity was estimated by person-hours from the reports maintained by NMMC and WHO and by interview with the NMMC and WHO staff involved. Also, interviews of the MO at each UHP and other UHP personnel who were personally involved in the activities was conducted to estimate their time and the time of other participants who could not be interviewed (e.g., ASHAs and drivers were not interviewed directly but their time was reported by the UHP personnel who supervised them). Participants in large trainings were assumed to have participated in the full length of the training, with an average roundtrip transport time per day based on the training location.

**Table 1:** Resource inputs for each activity in financial and economic costing.

Resource input categories for each activity	Details of resource inputs included for financial cost	Details of resource inputs included for economic cost
**Personnel Costs**	• N/A	• Personnel cadre type • Personnel cost (Salary) • Personnel time spent
**Allowance Costs**	• Per diem, travel allowances, remunerations	• Per diem, travel allowances, remunerations
**Supply and Material Costs**	• Material and supplies • Refreshments • Brochure/ vaccination cards, etc.	• Material and supplies • Refreshments • Brochure/ vaccination cards • WHO training materials
**Other Direct Costs**	• Other TCV campaign-related financial cost (e.g., vehicle rental, fuel)	• WHO travel cost (rental vehicle cost, owned vehicle cost, vehicle maintenance cost, fuel, driver cost, travel per diem) • WHO organizational expenses • NMMC travel cost (travel time, rental vehicle cost, owned vehicle cost, vehicle maintenance cost, fuel, driver cost, travel per diem) • UHP vehicle cost (travel time, rental vehicle cost)

### Data analysis

For travel costs, vehicle use by activity was estimated based on the interview with NMMC, WHO, and UHP staff who personally used the vehicles or observed the vehicles used by other personnel. For NMMC and UHP travel costs, existing vehicles were used, except for one UHP that hired an additional auto-rickshaw for the campaign outreach sessions. For WHO travel costs, all vehicles except one were rented via monthly contracts, from which a daily rate inclusive of driver, fuel, and insurance was calculated; the value of the WHO vehicle was annualized using a 3% discount rate. Service delivery costs were estimated for the three strategies used: fixed-site vaccination in UHPs, outreach sessions, and mop-up sessions. Wastage rates for vaccines and syringes were based on actual numbers of vaccine doses and syringes wasted as reported by all 11 UHPs by delivery strategy (fixed, outreach, and mop-up) from NMMC administrative records. Personnel costs for outreach and mop-up sessions were estimated by multiplying the number of sessions by the per diem per vaccination team, each of which consisted of 1 Auxiliary Nurse Midwife (ANM) and 3 Accredited Social Health Activists (ASHAs).

The financial and economic cost of TCV delivery was estimated in three sampled UHPs and at the NMMC level for activities that benefited all 11 UHPs. The total delivery cost for a UHP included the common NMMC-level delivery cost and the UHP-level TCV delivery cost that varied by each type of UHP. To calculate the total weighted average cost of the TCV campaign for 11 UHPs, the resources used at UHP-level for each activity were extrapolated from the three sampled UHPs to other remaining 8 UHPs of the same residential type in the catchment area (high-rise, slum, and mixed), then divided by the total doses to calculate the weighted average delivery cost per dose. Total financial and economic costs were estimated by program activity, level, payer, and per dose administered based on administrative coverage.

## Results

Across all 11 UHPs included in NMMC’s TCV campaign, the estimated total population of eligible children (9 months up to 15 years) was 159,831 based on prior polio vaccination campaign microplans and coverage, and the total number of TCV doses administered was 113,420, giving administrative coverage of 71.0% (Table 2). Out of the total 113,420 doses administered during the campaign, 2.4% were in high-rise UHPs, 71.3% inmixed UHPs, and 26.4% in slum UHPs. The administrative vaccination coverage rates in the three sampled UHPs were 46.5% (high-rise area), 70.9% (mixed area), and 76.1% (slum area) respectively. The total number of vaccination sessions and types of vaccination strategies differed by population size and characteristics of the residential area (Table 2).

**Table 2:** Vaccination coverage and strategies used in slum, mixed residence, and high-rise urban health posts (UHPs) during the Navi Mumbai Municipal Corporation (NMMC) typhoid conjugate vaccine (TCV) campaign, 2018.

	NMMC–vaccination campaign (11 UHPs)	UHP- Indiranagar (Majority slum UHP)	UHP- Ghansoli (Mixed residence UHP)	UHP- Sector 48 (High-rise UHP)
Total Children 9 months <15 years Eligible*	159,831	8,975	27,557	5,751
**Total Doses Administered**	113,420	6,832	19,528	2,673
**Total Vaccination Coverage %**	71.0%	76.1%	70.9%	46.5%
*Vaccination Strategies*				
**Fixed Sessions (%)**		8 (10.3%)	28 (10.4%)	17 (15.7%)
**Outreach/ Mop-up Sessions (%)**		69 (89.6%)	241 (89.6%)	91 (84.3%)
**Total Vaccination Sessions %)**		77 (100%)	269 (100%)	108 (100%)

*Based on microplan and coverage from polio vaccination campaigns in 2017–2018.

The total number of vaccines procured for the vaccination campaign was 116,880 and the total number of vaccines administered was 113,420 (Table 3). The number of vaccines procured was less than the total target population due to a phased procurement approach in which an initial volume was procured with potential for further real-time delivery from the supplier depending on uptake during the initial campaign dates; given the initial uptake rates, further procurement during the campaign beyond the initial volume was determined to be unnecessary. The market cost of the TCV vaccine (5-dose vial) procured was $2.93 (200.00 INR) and 36% of the vaccines were subsidized via donation of a share of doses by the distributor Bharat Biotech. A total of 118,570 injection syringes were procured and 113,420 were used, all of which were donated by the Government of India. The safety boxes were purchased by NMMC for $7.07 (483.00 INR) per unit and 150 units were distributed to 11 UHPs (Table 3).

**Table 3:** Vaccines and supplies used in during the Navi Mumbai Municipal Corporation (NMMC) typhoid conjugate vaccine (TCV) campaign, 2018, in 2018 USD (2018 INR).

	Vaccine	Injection syringe	Safety boxes
Total Number Procured	116,880	118,570	150
Total Number Administered	113,420	113,420	150
Total Number Wasted	3,460	5,150	0
% Wasted/Administered	3.1%	4.5%	0%
Market Cost (per dose/unit)	$2.93	$0.02	$7.07
	(200.00)	(1.69)	(483.00)
Subsidies (per dose/unit)	$1.07	$0.02	0
	(73.00)	(1.69)	
Subsidies % (per dose/unit)*	36%	100%	0%
Additional Costs % (per dose/unit)	n/a	5.3%*	n/a
Financial Cost (per dose/unit)	$1.86	<$0.01*	$7.07
	(127.00)	(0.09)	(483.00)
Economic Cost (per dose/unit)	$2.93	$0.03	$7.07
	(200.00)	(1.79)	(483.00)

* The vaccine was procured by NMMC from the manufacturer (Bharat Biotech), with 36% of the TCV doses donated free of charge by the manufacturer. Syringes were provided by the Government of India but the additional transportation costs from New Delhi to NMMC were covered by NMMC. NMMC procured safety boxes for the campaign for all participating UHPs.

The financial and economic costs per dose administered by program activity and level (NMMC or UHP) are shown in Table 4. Across all program activities and levels, the highest financial and economic cost per dose was incurred for purchase of vaccine and vaccination supplies at NMMC level, at $1.87 (127.70 INR) and $2.96 (202.40 INR), respectively. At UHP level, social mobilization and service delivery were the main cost drivers for vaccine delivery. The high-rise area UHP had the highest difference between total financial and economic delivery costs, with an economic cost per dose of $3.08 (210.46 INR) that was more than ten times higher compared to a financial cost per dose of $0.22 (14.94 INR).

**Table 4:** Financial and economic delivery costs per dose administered during the Navi Mumbai Municipal Corporation (NMMC) typhoid conjugate vaccine (TCV) campaign, 2018, by program activity in 2018 USD (2018 INR).

	NMMC-level costs		UHP- Indiranagar (Majority slum UHP)		UHP- Ghansoli (Mixed residence UHP)		UHP- Sector 48 (High-rise UHP)		Total weighted average cost of UHP level (11 UHPs) and NMMC cost	
**Number of TCV doses administered**	113,420		6,832		19,528		2,673		113,420	
**Activity**	Financial cost per dose-USD (INR)*	Economic cost per dose-USD (INR)*	Financial cost per dose-USD (INR)*	Economic cost per dose-USD (INR)*	Financial cost per dose-USD (INR)*	Economic cost per dose-USD (INR)*	Financial cost per dose-USD (INR)*	Economic cost per dose-USD (INR)*	Financial cost per dose-USD (INR)*	Economic cost per dose-USD (INR)*
**Planning and Preparation**	<$0.01 (0.07)	$0.05 (3.24)	0	<$0.01 (0.16)	0	0	0	$0.06 (4.43)	<$0.01 (0.10)	$0.05 (3.35)
**Microplanning**	<$0.01 (0.07)	$0.02 (1.60)	0	$0.03 (2.08)	0	$0.04 (2.44)	0	$0.18 (12.50)	<$0.01 (0.10)	$0.06 (4.18)
**Training**	0	$0.30 (20.24)	0	<$0.01 (0.95)	0	<$0.01 (0.29)	<$0.01 (0.19)	$0.04 (2.58)	<$0.01 (<0.01)	$0.30 (20.72)
**Sensitization^†^**	0	<$0.01 (0.47)	<$0.01 (0.53)	$0.14 (9.19)	<$0.01 (0.10)	$0.06 (4.35)	0	$0.12 (8.44)	<$0.01 (0.21)	$0.09 (6.22)
**Social Mobilization^†^**	$0.22 (14.80)	$0.25 (16.70)	<$0.01 (0.10)	$0.11 (7.75)	<$0.01 (0.44)	$0.14 (9.25)	<$0.01 (0.41)	$0.48 (32.44)	$0.22 (15.15)	$0.38 (26.10)
**Service Delivery (excluding vaccine, syringes, and safety boxes)**	$0.09 (6.07)	$0.09 (6.07)	$0.05 (3.30)	$0.17 (11.44)	$0.06 (3.86)	$0.17 (11.27)	$0.16 (10.84)	$1.54 (104.93)	$0.15 (9.97)	$0.29 (19.62)
**Supervision and Monitoring**	<$0.01 (0.19)	$0.07 (4.61)	0	$0.15 (10.17)	0	$0.02 (1.21)	$0.04 (2.99)	$0.44 (30.32)	$0.04 (0.27)	$0.13 (8.86)
**AEFI Preparedness and Management**	0	$0.12 (8.51)	<$0.01 (0.16)	$0.07 (4.84)	<$0.01 (0.14)	$0.05 (3.56)	$0.01 (0.51)	$0.22 (14.82)	<$0.01 (0.16)	$0.19 (12.67)
**Vaccine, syringe, and safety boxes**	$1.87 (127.70)	$2.96 (202.40)	n/a	n/a	n/a	n/a	n/a	n/a	$1.87 (127.70)	$2.96 (202.40)
**Total delivery cost (excluding vaccine, syringes, and safety boxes)**	$0.31 (21.20)	$0.90 (61.44)	$0.06 (4.09)	$0.68 (46.57)	$0.07 (4.54)	$0.47 (32.38)	$0.22 (14.94)	$3.08 (210.46)	$0.38 (25.86)	$1.49 (101.76)
**Total cost (including vaccine, syringes, and safety boxes)**	$2.18 (148.90)	$3.86 (263.84)							$2.25 (153.63)	$4.45 (304.16)

*2018 currency conversion 1 US$ = 68.30 INR [15] ^†^The cost to the WHO India Country Office of hiring a private public relations firm for social mobilization materials development and media monitoring and engagement was excluded from the analysis. In sensitivity analysis, the value of this contract would add US$0.09 to the economic cost per dose at NMMC-level and to the total weighted average of UHP and NMMC cost.

When combining NMMC-level and UHP-level costs, the total delivery cost per dose was highest in the high-rise UHP for both financial and economic costs [$0.53 (36.14 INR) and $3.98 (271.90 INR), respectively, excluding vaccine, syringe, and safety box costs] (Table 5). Excluding vaccine, syringe, and safety box costs, the lowest total financial cost per dose was in the mixed area UHP with $0.37 (25.30 INR) and the lowest total economic cost per dose was in the slum area UHP with $1.37 (93.8 INR).

**Table 5:** Total financial and economic delivery costs per dose administered during the Navi Mumbai Municipal Corporation (NMMC) typhoid conjugate vaccine (TCV) campaign, 2018, in 2018 USD (2018 INR).

	Majority slum UHP		Mixed residence UHP		High-rise UHP		Total weighted average cost of UHP level (11 UHPs)	
	Financial Cost per Dose- INR (USD)*	Economic Cost per Dose- INR (USD)*	Financial Cost per Dose- INR (USD)*	Economic Cost per Dose- INR (USD)*	Financial Cost per Dose- INR (USD)*	Economic Cost per Dose- INR (USD)*	Financial Cost per Dose- INR (USD)*	Economic Cost per Dose- INR (USD)*
**UHP-level delivery cost (not including vaccine, syringes, and safety boxes)**	$0.06 (4.09)	$0.68 (46.57)	$0.07 (4.54)	$0.47 (32.38)	$0.22 (14.94)	$3.08 (210.46)	$0.07 (4.66)	$0.59 (40.32)
**NMMC-level delivery cost (not including vaccine, syringes, and safety boxes)**	$0.31 (21.20)	$0.90 (61.44)	$0.31 (21.20)	$0.90 (61.44)	$0.31 (21.20)	$0.90 (61.44)	$0.31 (21.20)	$0.90 (61.44)
**Total delivery cost (not including vaccine, syringes, and safety boxes)**	$0.37 (25.29)	$1.58 (108.01)	$0.38 (25.74)	$1.37 (93.82)	$0.53 (36.14)	$3.98 (271.90)	$0.38 (25.86)	$1.49 (101.76)
**Total cost (including vaccine, syringes, and safety boxes)**	$2.24 (152.99)	$4.54 (310.40)	$2.25 (153.43)	$4.34 (296.20)	$2.40 (163.83)	$6.94 (474.30)	$2.25 (153.63)	$4.45 (304.16)

*2018 currency conversion 1 US$ = 68.30 INR

The total weighted average TCV delivery cost per dose including 11 UHPs and NMMC-level costs was $0.38 (25.86 INR) for financial cost and $1.49 (101.76 INR) for economic cost, excluding vaccine, syringe, and safety box costs. The much higher economic TCV delivery cost for high-rise UHP of $3.98 (271.90 INR) compared to other slum and mixed UHPs results from higher involvement and cost of human resources for social mobilization, microplanning, and service delivery activities. Some activities were implemented differently across UHPs; for example, financial costs for refreshments were incurred by the high-rise UHP for their training of social mobilizers but not for the mixed and slum UHPs’ analogous trainings, and for the mixed and slum UHPs’ sensitization meetings with local officials but not for those of the high-rise UHP. The total TCV campaign delivery cost per dose including vaccine, syringes, and safety boxes cost ranged across UHP types from $2.24–2.40 (152.99–163.83 INR) for the financial cost and $4.34–6.94 (296.20–474.30 INR) for the economic cost (Table 5 and S2 Table).

## Discussion

This study estimated the financial and economic delivery cost of the first-ever public sector TCV vaccine introduction in Navi Mumbai, India in 2018. The weighted average delivery cost per dose was estimated to be $0.38 for financial cost and $1.49 for economic cost, excluding the vaccine and vaccination supplies cost. As the first public sector TCV campaign conducted globally, this delivery costing study, alongside vaccine effectiveness, community surveys, and other surveillance and economic burden evaluations, provides empirical evidence that can help inform future TCV introduction in India and elsewhere. The Excel-based TCV Delivery Costing Tool developed and used here can be deployed for collecting and estimating TCV delivery costs elsewhere (S1 Tool).

The administrative coverage achieved varied among the different types of UHPs. The reported coverage was highest in slum areas and lowest in high-rise areas, which is likely due to the preference for private healthcare among the higher-income residents of the high-rise area [14]. Anecdotal reports from UHP staff suggested that high-income families had already vaccinated their children with TCV, which was previously only available in private clinics, leaving a smaller population of unvaccinated children to reach through the campaign in high-rise areas. The high overall coverage of 71% reflects NMMC’s effective strategy of planning the TCV campaign over weekends and public holidays to avoid the disruption of routine services, with vaccines given to children who had missed the dose through the routine immunization sessions [14]. Further mop-up activities were conducted to reduce the number of children who missed the TCV vaccination.

Also, it was reported anecdotally by health sector personnel that there was higher vaccine hesitancy in high-rise areas compared to slum and mixed areas. Therefore, additional efforts were required in planning and preparation, social mobilization, and sensitization activities. For example, the high-rise area UHP required more time and personnel for social mobilization activities like public loudspeaker announcements, community volunteers conducting house-to-house visits, and IEC activities. This also explains the highest financial and economic delivery cost for the high-rise UHP compared to other UHPs as more human resources and personnel were involved. Furthermore, the higher cost of the high-rise UHP can also be explained by the lower number of children vaccinated per session: 53 children on average, while in mixed and slum area UHPs, each session covered 102 and 117 children on average, respectively.

There are no existing studies on the estimated delivery cost of TCV that can be compared to this study. Published TCV cost-effectiveness analyses have assumed the full administrative and operational vaccination cost of TCV based on the country’s comprehensive Multi-Year Plan (cMYP) or used cost of delivery for routine immunization ($12.75 allocated per targeted child in 2018 prices) rather than using incremental micro-costing delivery cost data [6, 7]. However, there are existing studies on the delivery cost of different vaccines in India. One study on diphtheria–pertussis–tetanus (DPT) vaccine estimated an economic cost per dose of $2.36 (including vaccine costs in 2018 prices) at the national level and $1.42 to $2.93 at the state level based on data from 7 states in India (in 2018 prices) [16]. For oral cholera vaccine (OCV) vaccination in Odisha, India in 2011, the financial delivery cost was $0.49 per dose in 2011 prices, or $0.55 in 2018 prices [17]. However, the cholera vaccine is administrated orally and to all populations aged 1 and above, which is different from this TCV campaign. The TCV delivery cost presented here is comparable with the standardized country-level economic delivery cost per dose for routine childhood immunization services of $1.87 (95% uncertainty interval $0.64–4.38) in 24 countries (in 2018 prices) [18].

There are several limitations to this study. First, only three UHPs representing three different residential (slum, mixed, high-rise) areas were sampled purposively, which may not be representative of other UHPs in Navi Mumbai. Second, not all individuals who participated in the activities were interviewed during the data collection process; for some activities, information was provided by the responsible person who oversaw the implementation of the activity (e.g., time spent by each cadre of personnel in an activity). Although those who reported this information had a thorough knowledge of and involvement in the vaccination campaign activities, they may not have directly observed all activities performed by others; hence, personnel time, in particular, may be overestimated or underestimated as a result. Also, the average transport time for meetings and standard allowance was assumed for all participants in the meetings and trainings. The cost of vaccine delivery activities was calculated based on the number of mobile vaccination teams and the average per diem given to each team as we assumed that everyone on the vaccination teams received a standard per diem rate. Since we did not interview all individuals involved in the vaccination teams and individual payment records, the assumption of standard per diem may have overestimated or underestimated the actual cost. Third, some information reported by key personnel at NMMC and UHP levels may be subject to recall bias since the nationwide Measles-Rubella [19] vaccination campaign was conducted within the NMMC jurisdiction between the TCV campaign and the time of TCV campaign cost data collection. Research team has reviewed and collected data from NMMC and WHO financial and programmatic records to minimize recall bias. Finally, campaign vaccination coverage was estimated based on administrative data and estimates of the size of the target population derived from microplans and coverage estimates of recent polio vaccination campaigns, which may not be reliable.

## Conclusion

This study is the first costing study of a large-scale implementation of TCV within a public sector immunization program using a micro-costing approach. These findings are useful in estimating resource requirements for TCV campaigns in the future as well as to understand the value of TCV campaigns through economic evaluations in conjunction with other data. As WHO has developed new recommendations for TCV use and Gavi has included it in their vaccine portfolio, the results from this study will inform and guide not only local and national policies but also global decisions about the potential use of TCV as part of comprehensive typhoid prevention and control strategies. The experience and tools developed as a part of this study can be used to estimate TCV delivery costs in other settings.

## Supporting information

S1 Tool Typhoid conjugate vaccine delivery costing tool. (XLSX)

S1 Table Program activities included in cost analysis of the Navi Mumbai Municipal Corporation (NMMC) typhoid conjugate vaccine (TCV) campaign, 2018 [20]. (DOCX)

S2 Table Total financial and economic cost by activity during the Navi Mumbai Municipal Corporation (NMMC) typhoid conjugate vaccine (TCV) campaign, 2018 (in 2018 USD; INR). (DOCX)
