# Supplementary material for: Delivery cost of the first public sector introduction of typhoid conjugate vaccine in Navi Mumbai, India
Source: PLOS Glob Public Health. 2023 Jan 4;3(1):e0001396. doi: 10.1371/journal.pgph.0001396 (PMC10022355; doi:10.1371/journal.pgph.0001396)
Supplement: S1 Table — (DOCX) [file pgph.0001396.s002.docx]

**Title: Delivery Cost of the First Public Sector Introduction of Typhoid Conjugate Vaccine in Navi Mumbai, India**

Authors: Dayoung Song^1^, Sarah W. Pallas^2^, Rahul Shimpi^3^, N. Ramaswamy^4^, Pradeep Haldar^5^, Pauline Harvey^3^, Pankaj Bhatnagar^3^, Arun Katkar^3^, Niniya Jayaprasad^3^, Abhishek Kunwar^3^, Sunil Bahl^6^, Win Morgan^7^, Raymond Hutubessy^8^, Kashmira Date^2^, Vittal Mogasale^9*^

Author affiliations:

^1^Policy and Economic Research Department, International Vaccine Institute, Republic of Korea

^2^Global Immunization Division, Centers for Disease Control and Prevention, Atlanta, Georgia, USA

^3^World Health Organization, India Country Office, New Delhi, India

^4^Navi Mumbai Municipal Corporation, Navi Mumbai, India

^5^Ministry of Family Health and Welfare, Government of India, New Delhi, India

^6^World Health Organization, Regional Office for South-East Asia, New Delhi, India

^7^Levin and Morgan LLC, Bethesda, MD, USA

^8^Department of Immunization, Vaccines and Biologicals, World Health Organization, Geneva, Switzerland

Ministry of Health and Family Welfare, Government of India, India

^9^Policy and Economic Research Department, International Vaccine Institute, Republic of Korea (Current affiliation: Department of Health Systems Governance and Financing, World Health Organization, Geneva, Switzerland)

*Email: [vmogasale@gmail.com](mailto:vmogasale@gmail.com); [mogasalev@who.int](mailto:mogasalev@who.int)

**S1_Table. Program activities included in cost analysis of the Navi Mumbai Municipal Corporation (NMMC) typhoid conjugate vaccine (TCV) campaign, 2018**

| Main campaign activity category | Campaign activity sub-categories | Activities | Description |
| --- | --- | --- | --- |
| Planning and Preparation | Proposals and approvals to Committees | Proposal to general body committee | The vaccine was procured by NMMC from Bharat Biotech, with 36% of the TCV doses donated free of charge by the manufacturer. Syringes were provided by the Government of India but the additional transportation costs from New Delhi to NMMC was covered by NMMC. NMMC procured safety boxes for the campaign for all participating UHPs. |
|  |  | Proposal to Standing Committee |  |
|  |  | Approval of proposal by Standing Committee |  |
|  | Vaccine procurement and agreement | Agreement with Bharat Biotech for vaccine supply |  |
|  |  | Agreement from Bharat Biotech (iterations) |  |
|  |  | Vaccine order |  |
|  | Cold chain planning | Cold chain planning |  |
|  | Waste disposal | Planning of biomedical waste disposal |  |
|  | Technical committee meeting | Technical committee meeting |  |
| Microplanning | Finalization of microplanning tools | Finalization of microplanning tools |  |
|  | UHP microplanning | Microplan preparation at UHP |  |
|  |  | Submission of the microplan at HQ |  |
|  |  | Finalization of vaccinators and supervisors for campaign at UHP |  |
|  | Finalization of the microplan at headquarters (HQ) | Finalization of the microplan at HQ |  |
|  |  | Finalization of vaccinators and supervisors for campaign at HQ |  |
| Training | Finalization of training material | Finalization of training materials | Training sessions were conducted at both NMMC and UHP levels. Trainings conducted at NMMC were led by WHO-India staff and involved training of pediatricians, UHP medical officers, supervisors, and ANMs. The training sessions covered AEFI management, microplanning, campaign logistics and operations, injection techniques, and supervision. Training sessions at UHP level were led by UHP medical officers and covered training of UHP personnel, Accredited Social Health Activists (ASHAs) and community mobilizers. |
|  | Microplanning & AEFI Training at HQ | Microplanning & AEFI training at HQ (MO, supervisors, and Auxiliary Nurse Midwives (ANMs) |  |
|  |  | AEFI Training at HQ (MO, Pediatricians) |  |
|  | Training of mobilizers | Mobilizers (link workers/ASHAs) orientation at UHP |  |
|  | Operational Training at HQ | Operational training at HQ (how the campaign will be operationalized) |  |
| Sensitization^†^ | Stakeholder meeting/information sharing | Letter to WHO/State/Government of India | Focus group discussions (FGDs) were conducted with campaign workers, pediatricians, caregivers, and teachers, and the information was used in planning for campaign messaging. Stakeholder meetings were conducted in NMMC and UHP level. |
|  |  | Meeting with Honorable Mayor and Commissioner |  |
|  |  | Meeting with local corporators |  |
|  | Meeting with stakeholders (UHP Level) | Communication of plan with housing societies/other influencers for support |  |
|  |  | Communication/meeting with housing societies |  |
|  | Press & Media activities | Media Monitoring - WHO |  |
|  |  | Media briefing (Press Conference) |  |
|  |  | Press release (for all) |  |
| Social Mobilization^†^ | Focus group discussions | Orientation of FGD facilitators |  |
|  |  | Key messages/talking points developed for different stakeholders (FGD) | Target population enumeration and social mobilization were done by health facility staff and community volunteers in each targeted area in the week before the outreach session. Social mobilization activities included meetings with mothers’ groups, apartment building associations, distribution of leaflets and posters to homes and private health care providers in the area, and health facility staff circulating in neighborhoods with megaphones to announce the campaign during outreach days. |
|  |  | Key messages/talking points developed for different stakeholders (FGD) (UHP Level) |  |
|  | Information, education, and communication (IEC) activities | Finalization of frequently asked questions (FAQs) |  |
|  |  | IEC material prototyping |  |
|  |  | IEC material finalization |  |
|  |  | IEC material printing |  |
|  |  | IEC distribution to UHP |  |
|  | House-to-house pre-campaign identification of eligible children (TCV Pre-Campaign Survey) | ASHAs and other community volunteers went house to house to enumerate the children eligible for the campaign, distribute vaccination cards, and share information with parents and caregivers about the upcoming campaign |  |
|  | Inauguration | Inauguration of campaign |  |
| Service Delivery | Supply of syringes & needles to UHP | Vaccine and logistics; supply to UHP | The campaign delivered a single dose of injectable TCV (Typbar-TCV; Bharat Biotech International) to eligible children. The TCV campaign was conducted via outreach sessions on weekends and public holidays for 10 days over four weeks, followed by three mop-up days targeting areas with low administrative coverage, plus fixed-site vaccination sessions within UHPs on at least two weekdays per week during the campaign period. Vaccinations were administered by ANMs during outreach sessions, with each team composed of one ANM and ASHAs and other community volunteers to assist with social mobilization and registration. Fixed-site vaccinations were administered by a Nurse-Midwife (NM) as part of routine immunization activities. The cost of vaccine, syringes, and safety boxes were not included in costing of service delivery. |
|  |  | Supply of syringes & needles by GOI |  |
|  | Vaccination campaign | Vaccination campaign |  |
|  | Biomedical waste bags | Biomedical waste bags |  |
| Supervision and Monitoring | Supervision | Supervision | Supervision was conducted by NMMC staff and UHP MOs and Lady Health Volunteers (LHVs). Monitoring was provided by external monitors contracted by WHO-India. |
|  |  | Supervision (UHP Level) |  |
|  | Monitoring by WHO | Monitoring by WHO |  |
|  |  | Monitoring by WHO (UHP Level) |  |
|  | Briefing of WHO Monitors | Briefing of WHO Monitors |  |
|  | Debriefing of WHO Monitors with NMMC | Debriefing of WHO Monitors with NMMC |  |
| AEFI Preparedness and Management | AEFI Training | Orientation of Indian Academy of Pediatrics (IAP) members, office bearer of Indian Medical Association [20] & other professional organizations | Adverse events following immunization (AEFI) committees were formed for NMMC as a whole and within each UHP, composed of local doctors and other experts (e.g., social workers). Each UHP prepared an AEFI kit with essential medicines from NMMC central medical stores or local markets and monitored recipients for 30 minutes post-vaccination for any AEFI. AEFI-related trainings were conducted and AEFI activities in UHPs were supervised and monitored by WHO. |
|  |  | Orientation of IAP members, office bearer of IMA & other professional organizations (UHP Level) |  |
|  | AEFI committee orientation | Orientation of AEFI Committee |  |
|  |  | Orientation of AEFI Committee (UHP Level) |  |
|  | AEFI kit Preparation | Preparation of AEFI kits for Campaign |  |
|  | AEFI Communication | AEFI Phone call to medical officer (MO) |  |
| Abbreviations used: ASHA: Accredited Social Health Activists; ANM: Auxiliary Nurse Midwives; AEFI: Adverse Event Following Immunization; FAQs: frequently asked questions; FGD: focus group discussion; GOI: Government of India; HQ: headquarters; IAP: Indian Academy of Pediatrics; IEC: information, education, and communication; IMA: Indian Medical Association; LHV: Lady Health Volunteers; MO: Medical Officer; NM: Nurse Midwife; NMMC: Navi Mumbai Municipal Corporation; TCV: typhoid conjugate vaccine; WHO: World Health Organization; UHP: urban health post  ^†^The cost to the WHO India Country Office of hiring a private public relations firm for social mobilization materials development and media monitoring and engagement was excluded from the analysis. | | | |
