# Supplementary material for: Delivery cost of the first public sector introduction of typhoid conjugate vaccine in Navi Mumbai, India
Source: PLOS Glob Public Health. 2023 Jan 4;3(1):e0001396. doi: 10.1371/journal.pgph.0001396 (PMC10022355; doi:10.1371/journal.pgph.0001396)
Supplement: S2 Table — (DOCX) [file pgph.0001396.s003.docx]

**Title: Delivery Cost of the First Public Sector Introduction of Typhoid Conjugate Vaccine in Navi Mumbai, India**

Authors: Dayoung Song^1^, Sarah W. Pallas^2^, Rahul Shimpi^3^, N. Ramaswamy^4^, Pradeep Haldar^5^, Pauline Harvey^3^, Pankaj Bhatnagar^3^, Arun Katkar^3^, Niniya Jayaprasad^3^, Abhishek Kunwar^3^, Sunil Bahl^6^, Win Morgan^7^, Raymond Hutubessy^8^, Kashmira Date^2^, Vittal Mogasale^9*^

Author affiliations:

^1^Policy and Economic Research Department, International Vaccine Institute, Republic of Korea

^2^Global Immunization Division, Centers for Disease Control and Prevention, Atlanta, Georgia, USA

^3^World Health Organization, India Country Office, New Delhi, India

^4^Navi Mumbai Municipal Corporation, Navi Mumbai, India

^5^Ministry of Family Health and Welfare, Government of India, New Delhi, India

^6^World Health Organization, Regional Office for South-East Asia, New Delhi, India

^7^Levin and Morgan LLC, Bethesda, MD, USA

^8^Department of Immunization, Vaccines and Biologicals, World Health Organization, Geneva, Switzerland

Ministry of Health and Family Welfare, Government of India, India

^9^Policy and Economic Research Department, International Vaccine Institute, Republic of Korea (Current affiliation: Department of Health Systems Governance and Financing, World Health Organization, Geneva, Switzerland)

*Email: [vmogasale@gmail.com](mailto:vmogasale@gmail.com); [mogasalev@who.int](mailto:mogasalev@who.int)

**S2_Table. Total financial and economic cost by activity during the Navi Mumbai Municipal Corporation (NMMC) typhoid conjugate vaccine (TCV) campaign, 2018 (in 2018 USD; INR)**

|  | NMMC-level costs | | Majority slum UHP | | Mixed residence UHP | | High-rise UHP | |
| --- | --- | --- | --- | --- | --- | --- | --- | --- |
| Number of TCV doses administered | 113,420 | | 6,832 | | 19,528 | | 2,673 | |
| Activity | Total Financial Cost - USD (INR)* | Total Economic Cost - USD (INR)* | Total Financial Cost - USD (INR)* | Total Economic Cost - USD (INR)* | Total Financial Cost - USD (INR)* | Total Economic Cost - USD (INR)* | Total Financial Cost - USD (INR)* | Total Economic Cost - USD (INR)* |
| Planning and Preparation | $117.13  (8,000) | $5,387.36  (367,957) | 0 | $16.12  (1,101) | 0 | 0 | 0 | $173.35 (11,840) |
| Microplanning | $117.11  (8,000) | $2,653.69  (181,247) | 0 | $207.85  (14,196) | 0 | $699.02  (47,743) | 0 | $489.21 (33,413) |
| Training | 0 | $33,607.10  (2,295,365) | 0 | $94.66  (6,465) | 0 | $82.23  (5,616) | $7.32  (500) | $101.11 (6,906) |
| Sensitization^†^ | 0 | $ 785.97  (53,682) | $52.71  (3,600) | $ 916.13 (62,572) | $29.28  (2,000) | $1,244.86  (85,024) | 0 | $330.31  (22,560) |
| Social Mobilization^†^ | $24,572.37  (1,678,293) | $ 27,707.42 (1,892,417) | $10.07  (688) | $774.83 (52,921) | $124.60 (8,510) | $2,645.62 (180,696) | $16.11  (1,100) | $ 1,269.44 (86,703) |
| Service Delivery | $ 10,087.50  (688,976) | $ 10,087.50  (688,976) | $330.28 (22,558) | $1,144.19 (78,148) | $ 1,102.49 (75,300) | $ 3,222.14 (220,072) | $ 131.41 (8,975) | $4,106.65 (280,484) |
| Supervision and Monitoring | $ 314.06  (21,450) | $ 7,654.63 (522,811) | 0 | $1,017.73 (69,511) | 0 | $344.76 (23,547) | $117.13  (8,000) | $1,186.65  (81,048) |
| AEFI Preparedness and Management | 0 | $ 14,136.37 (965,514) | $15.72  (1,074) | $484.29 (33,077) | $41.29  (2,820) | $ 701.54 (47,915) | $19.77  (1,350) | $579.90  (39,607) |
| Vaccine, syringe, and safety boxes | $ 218,405.02  (14,917,063) | $ 218,405.02  (23,648,702) | n/a | n/a | n/a | n/a | n/a | n/a |
| Total cost (including vaccine, syringes, and safety boxes) | $ 253,613.21  (17,321,782) | $ 448,267.51  (30,616,671) |  |  |  |  |  |  |
| Total delivery cost (not including vaccine, syringes, and safety boxes) | $ 35,208.18  (2,404,719) | $ 102,020.04 (6,967,969) | $408.78 (27,920) | $ 4,658.43 (318,171) | $1,297.66  (88,630) | $ 9,257.67  (632,299) | $584.55  (39,925) | $ 8,236.62  (562,561) |

*2018 currency conversion 1 US$= 68.30 INR ^†^The cost to the WHO India Country Office of hiring a private public relations firm for social mobilization materials development and media monitoring and engagement was excluded from the analysis. The value of this contract would add US$9,868.23 to the total economic cost at NMMC-level
